# Air Pollution and Serum C-reactive Protein Concentration in Children

**DOI:** 10.2188/jea.17.169

**Published:** 2007-09-01

**Authors:** Masayuki Shima

**Affiliations:** 1 Department of Public Health, Hyogo College of Medicine.

**Keywords:** C-reactive protein, Air pollution, Suspended particulate matter (SPM), Asthma, Wheezing

## Abstract

**BACKGROUND:**

Few biological markers that allow evaluation of the effects of air pollution on human health have been identified. This study evaluated the association of serum C-reactive protein (CRP) concentration in children with their respiratory symptoms and air pollution.

**METHODS:**

Respiratory symptoms and serum concentrations of CRP were examined in 2,094 school children living in 3 communities with different concentrations of air pollutants in Chiba Prefecture, Japan in 2001. The relationships between serum CRP concentration and sex, age, respiratory symptoms, and various environmental factors were analyzed.

**RESULTS:**

Serum CRP concentration decreased with age, and was significantly higher both in children who were bottle-fed in infancy and whose mothers smoked. Children with wheeze had significantly higher serum CRP concentration than those without wheeze. After adjustment for potential confounding factors, increased serum CRP concentrations of the 90th percentile (1.4 mg/L) or above were significantly associated with atmospheric concentration of suspended particulate matter (SPM) (odds ratio [OR] =1.49 for the range of observed concentrations, 95% confidence interval [Cl]: 1.07-2.06) and sulfur dioxide (SO_2_) (OR =1.45, 95% Cl: 1.04-2.03). In a two-pollutant model including SPM and nitrogen dioxide (NO_2_) concentrations, increased serum CRP concentrations were also associated with SPM (OR =1.94, 95% Cl: 1.08-3.50), but no such association was found with NO_2_ (OR =0.62, 95% Cl: 0.26-1.48).

**CONCLUSION:**

Serum CRP concentration is related to wheezing and the degree of air pollution. Because the concentrations of air pollutants are highly correlated, it is difficult to elaborate on which pollutant has a stronger effect on serum CRP concentrations.

The concentrations of air pollutants, represented by suspended particulate matter (SPM) and nitrogen dioxide (NO_2_), have increased due to the increase in motor vehicle traffic in urban areas in Japan.^1^ The effects of such air pollution on human health become of major concern. For children, most epidemiologic studies have focused on the effect of air pollution on respiratory symptoms or disease.^2^^-^^4^ There has long been a need for sensitive biomarkers to objectively evaluate the chronic effects of air pollution on human health as early as possible, but none has yet been identified.^5^^,^^6^ Previously, we reported an association between serum concentrations of acute phase proteins or hyaluronate in children and concentrations of air pollutants,^7^^,^^8^ but assays for these serum factors have not been validated as biomarkers for the effects of air pollution.

C-reactive protein (CRP) in serum has been widely used as a marker for infectious disease.^9^ A population-based study in Finland reported the association of serum CRP concentration with bronchial asthma.^10^ Because high-sensitivity tests of CRP are now available, it is possible to detect slight inflammatory conditions or local tissue damage that would have been undetectable by previous methods.^11^ Serum CRP concentration has been reported to be useful for prediction of cardiovascular disease,^12^^,^^13^ because such concentrations have been associated with atherosclerosis. Furthermore, acute effects of particulate air pollution on serum CRP concentration have been previously documented in adults.^14^^-^^18^ Although the acute effect of air pollution on serum CRP has also been observed in healthy young people,^19^ there are no reported studies of the association between serum CRP concentration and chronic exposure to air pollution. Therefore, the author conducted a cross-sectional study in 3 communities with different concentrations of air pollutants, and measured serum CRP concentration in school children. The levels of serum CRP were examined for association with sex, age, respiratory symptoms, and various environmental factors.

## METHODS

### Study Population

The study population consisted of the entire children (2,540 pupils, grades 1-6, aged 6-12 years) attending 6 elementary schools from 3 different communities in Chiba Prefecture, Japan (Figure). Of these schools, three were in an urban district next to the Tokyo metropolitan area (Ichikawa), and located near major roads. The distance between a school and a road ranged from 50 to 700 m. Of the other schools, one was located in the interior of the Boso Peninsula (Obitsu) and two were adjacent to the coast of Tokyo Bay (Kimitsu). There were no major roads near these three schools.

**Figure.**
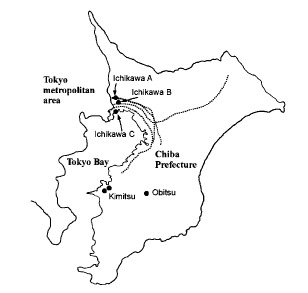
Location of study schools and major trunk roads in Chiba Prefecture, Japan. The dotted lines show major trunk roads.

### Air Pollution Measurements

Air pollutants are monitored continuously by the local authorities of the Chiba Prefectural Government. In Japan, particulate air pollution is assessed based on the concentration of SPM, which is the fraction of particles with a diameter less than 10 µm. The usual concentration of SPM corresponds approximately to that of particles passing an inlet with 50% cut-off efficacy using an aerodynamic diameter of 7 µm (PM_7_).^20^ The average concentrations of SPM, NO_2_, and sulfur dioxide (SO_2_) for the 3-year period from 1998 through 2000, measured at monitoring stations proximal to the study schools, are shown in Table 1. The distance between a school and a monitoring station ranged from 0 to 1.4 km. All concentrations measured were lowest in Obitsu. The concentrations in Kimitsu, which is located in an industrial area, were moderately high. All 3 stations in Ichikawa, which has heavy motor vehicle traffic, had the highest concentrations of air pollutants. The concentrations of air pollutants did not change substantially during the study period. The correlation between SPM and NO_2_ was high (correlation coefficient = 0.86). The concentration of SO_2_ also showed very strong correlation with SPM and NO_2_ (correlation coefficients = 0.99 and 0.92, respectively).

**Table 1.** Average concentrations* of atmospheric air pollutants in the study communities.

	SPM (µg/m^3^) Mean (SD)	NO_2_ (ppb) Mean (SD)	SO_2_ (ppb) Mean (SD)
Ichikawa (School A)	41.7 (3.2)	25.3 (2.5)	6.3 (0.6)
Ichikawa (School B)	42.7 (2.9)	23.7 (1.5)	6.3 (0.6)
Ichikawa (School C)	40.3 (6.5)	29.0 (2.0)	6.3 (1.5)
Kimitsu	28.0 (1.0)	19.0 (1.0)	4.7 (1.2)
Obitsu	26.7 (2.5)	11.7 (2.1)	4.3 (0.6)

*: Data are shown as the mean values (SD) of the annual average concentrations of air pollutants for the 3 year period 1998-2000, measured at ambient air monitoring stations located near the study schools.

SPM: Suspended particulate matter

NO_2_: Nitrogen dioxide

SO_2_: Sulfur dioxide

ppb: parts per billion

SD: Standard deviation

### Data Collection, Blood Sampling, and Laboratory Measurements

School children were examined in October 2001 using a standard respiratory symptom questionnaire, the modified Japanese version of ATS-DLD-78-C.^21^ The questionnaires were distributed through their schools and completed by either parents or guardians. Improperly completed questionnaires were returned to the appropriate individuals in order to obtain complete information. Based on replies to the questionnaire, children who had 2 or more past episodes of wheezing accompanied by dyspnea, and who had also experienced asthmatic attacks or the need for any medical treatment for asthma by physicians in the previous 2 years, were classified as having asthma. Children who had 2 or more wheezing episodes in the previous 2 years without a history of asthma were classified as having wheeze. Questions concerning the presence of smokers in the family and feeding methods in infancy were also included in the questionnaire.

Blood samples were collected between October and November 2001 from children for whom written consent had been obtained from their parents or guardians. Children with signs of acute inflammation, such as a cold or fever, were excluded from the present study. The collected blood samples were centrifuged on the same day and concentrations of total IgE and CRP in the serum were determined using latex-enhanced immunonephelometric assays on a BN II analyzer (Dade Behring, Marburg, Germany). The minimum detection level of CRP was 0.2 mg/L.^22^

### Data Analyses

The serum concentrations of CRP were distributed approximately log normally.^23^ These values were therefore converted to logarithms for analysis, and the results expressed as geometric means with 95% confidence intervals (CIs). The minimum detection limit of the assay (0.2 mg/dL) was used as the value for undetectable CRP level in the calculation of geometric means. Data were compared according to the differences in the following factors: sex, age, feeding methods in infancy, smoking habits of the family, asthma or wheezing. Serum IgE concentration was used as a marker for predisposition to atopy. The serum CRP concentration was compared between children with serum IgE concentrations of 250 IU/mL or greater and those with concentrations below 250 IU/mL, as reported in a previous report.^7^

Because the serum CRP concentration was undetectable among more than half the children, the concentrations were then dichotomized at the 90th percentile (1.4 mg/L), and the prevalence of a serum CRP concentration of 1.4 mg/L or above was compared in relation to various factors. To evaluate the effects of each factor, multiple logistic regression analyses were performed using the high serum CRP concentrations as a binary outcome. The 3-year average concentrations of air pollutants in the communities where the children lived were included in the models as continuous variables. Statistical analyses were performed using SPSS^®^ software (SPSS Inc., Chicago, IL, USA).

## RESULTS

Questionnaires were collected from 2,501 children (98.5%), blood samples were collected from 2,097 children (82.6%), and useable data for both were obtained from 2,094 children (82.4%). The characteristics of the study children are shown according to study communities in Table 2. There were no differences in sex, age, asthma or wheezing, or serum IgE concentrations in the children from the 3 communities. The proportion of bottle-fed children was higher in Obitsu than in other communities, while that of breast-fed children was higher in Ichikawa. However, the proportion of mixed-fed children was similar among the 3 communities. The proportion of smoking mothers in Obitsu was low. In contrast, the proportion of family members who were smokers, other than mothers, was significantly higher in Obitsu than in other communities.

**Table 2.** Demographics and health characteristics (%) of the study subjects, by community.

	Ichikawa (n = 1009)	Kimitsu (n = 746)	Obitsu (n = 339)	p value
Sex				
Male	50.0	52.5	51.6	0.577
Female	50.0	47.5	48.4	
Age (year)				
6-7	22.2	26.1	22.1	0.227
8-10	49.1	47.7	46.9	
11-12	28.7	26.1	31.0	
Mean age (years)	9.19	9.02	9.24	0.068
(SD)	(1.79)	(1.83)	(1.79)	
Feeding method in infancy				
Bottle	18.2	26.5	36.6	<0.001
Breast	34.9	25.7	18.3	
Mixed	46.9	47.7	45.1	
Familial smoking habits				
Mother smokes	18.3	21.7	11.8	<0.001
Others smoke	40.1	44.4	56.9	
No one smokes	41.5	33.9	31.3	
Respiratory symptoms				
Wheezing	6.0	7.1	4.4	0.292
Asthma	8.4	8.0	6.2	
No symptoms	85.5	84.9	89.4	
Serum IgE concentration				
0-249 IU/mL	69.8	69.4	65.8	0.37
≥ 250 IU/mL	30.2	30.6	34.2	

SD : Standard deviation

Table 3 shows the results of univariate analysis of serum CRP concentration and the prevalence of a serum CRP concentration of the 90th percentile (1.4 mg/L) or above, according to sex, age, feeding method in infancy, familial smoking habits, respiratory symptoms, serum IgE concentrations, and study communities. The serum CRP concentration was highest in the youngest children (6-7 years), and decreased with age. The prevalence of a serum CRP concentration of the 90th percentile or above was highest in children aged 6-7 years. Children who were bottle-fed in infancy had significantly higher serum CRP concentrations than children who were either breast- or mixed-fed. The serum CRP concentration in children whose mothers were smokers was significantly higher than in children without smokers in the family. The smoking habits of family members other than mothers were not significantly related to serum CRP concentration. The serum CRP concentration in children with wheeze was higher than in children without wheeze. The prevalence of a serum CRP concentration of the 90th percentile or above was also higher in children with wheeze. Children with asthma had higher serum CRP concentrations than those without, but the difference was not statistically significant. Analysis of serum CRP concentration according to study community revealed that the concentrations were highest in Ichikawa among the three communities, followed by Kimitsu, and then Obitsu, however these differences were not statistically significant. The differences in serum CRP concentration according to sex and serum IgE concentrations were not statistically significant.

**Table 3.** Univariate analysis of serum C-reactive protein (CRP) concentration (mg/L) and prevalence (%) of CRP ≥ the 90th percentile (1.4 mg/L) in children relative to various factors.

	n	Geometric mean (mg/L)			CRP ≥ 1.4 mg/L	
	
			(95% CI)	p value	%	p value
Sex						
Male	1072	0.36	(0.34-0.38)	0.349	10.5	0.619
Female	1022	0.35	(0.33-0.37)		9.9	
Age (years)						
6-7	494	0.46	(0.42-0.51)	<0.001	16.4	<0.001
8-10	1011	0.34*	(0.32-0.36)		9.7	
11-12	589	0.29^*,†^	(0.27-0.31)		5.9	
Feeding method in infancy						
Bottle	506	0.4	(0.36-0.44)	0.002	13.8	0.009
Breast	606	0.34^‡^	(0.32-0.37)		8.9	
Mixed	982	0.34^§^	(0.32-0.35)		9.2	
Familial smoking habits						
Mother smokes	387	0.41	(0.37-0.46)	<0.001	14.2	0.014
Others smoke	929	0.35^||^	(0.33-0.37)		9.7	
No one smokes	778	0.33^||^	(0.31-0.35)		8.9	
Respiratory symptoms						
Wheezing	129	0.47	(0.38-0.57)	<0.001	17.8	0.002
Asthma	166	0.41	(0.35-0.47)		13.9	
No symptoms	1799	0.34^¶^	(0.33-0.36)		9.3	
Serum IgE concentration						
0-249 IU/mL	1445	0.35	(0.33-0.37)	0.475	10.1	0.794
≥ 250 IU/mL	649	0.36	(0.33-0.39)		10.5	
Study community						
Ichikawa	1009	0.36	(0.34-0.38)	0.483	11.3	0.259
Kimitsu	746	0.35	(0.33-0.37)		9.5	
Obitsu	339	0.34	(0.31-0.37)		8.6	

The significance of the differences in serum CRP concentration among the groups was evaluated by Tukey's method.

* : p<0.01 compared with children 6-7 years old.

† : p<0.01 compared with children 8-10 years old.

‡ : p<0.05, § : p<0.01 compared with children who were bottle-fed in infancy.

|| : p<0.01 compared with children whose mothers smoke.

¶ : p<0.01 compared with children with wheeze.

CI : confidence interval

The effects of various factors on serum CRP concentration of the 90th percentile or above were analyzed using logistic regression analysis (Table 4). The adjusted odds ratio (OR) of high serum CRP concentration for age was significantly below one. High serum CRP concentration was also significantly associated with bottle-feeding in infancy, maternal smoking habit and wheezing. The association of high serum CRP concentration with SPM concentration in the communities where the children lived was significant (OR = 1.49 for the range of observed concentrations, 95% CI: 1.07-2.06). High serum CRP concentration was also associated with SO_2_ concentration (OR = 1.45 for the range of observed concentrations, 95% CI: 1.04-2.03), but the association with NO_2_ concentration was not statistically significant (OR = 1.41 for the range of observed concentrations, 95% CI: 0.87-2.28). In a two-pollutant model including SPM and NO_2_ concentrations, high serum CRP concentration was also significantly associated with SPM (OR = 1.94 for the range of observed concentrations, 95% CI: 1.08-3.50), but no such association was found with NO_2_ (OR = 0.62 for the range of observed concentrations, 95% CI: 0.26-1.48). Because SO_2_ concentration strongly correlated with SPM and NO_2_, two-pollutant models including SO_2_ concentration were not analyzed.

**Table 4.** Adjusted odds ratios (ORs)* and 95% confidence intervals (CIs) for various factors on C-reactive protein (CRP) ≥ the 90th percentile (1.4 mg/L).

	A single-pollutant model including SPM concentration		A single-pollutant model including NO_2_ concentration		A single-pollutant model including SO_2_ concentration		A two-pollutant model including SPM and NO_2_ concentrations	
			
	OR (95% CI)	p value	OR (95% CI)	p value	OR (95% CI)	p value	OR (95% CI)	p value
Age (1 year increase)	0.83 (0.76-0.90)	<0.001	0.83 (0.77-0.90)	<0.001	0.83 (0.76-0.90)	<0.001	0.83 (0.76-0.90)	<0.001
Sex (females vs. males)	0.92 (0.69-1.23)	0.581	0.93 (0.70-1.24)	0.621	0.92 (0.69-1.24)	0.594	0.92 (0.69-1.23)	0.562
Bottle-fed	1.67 (1.22-2.28)	0.001	1.63 (1.19-2.23)	0.002	1.66 (1.22-2.27)	0.001	1.65 (1.21-2.26)	0.002
Maternal smoking	1.48 (1.06-2.07)	0.023	1.47 (1.05-2.06)	0.024	1.48 (1.06-2.07)	0.023	1.49 (1.06-2.08)	0.021
Serum IgE concentration (≥ 250 IU/mL vs. 0-249 IU/mL)	1.09 (0.77-1.53)	0.624	1.09 (0.78-1.53)	0.619	1.09 (0.77-1.53)	0.621	1.09 (0.77-1.53)	0.628
Respiratory symptoms								
Wheezing	1.85 (1.11-3.08)	0.018	1.84 (1.11-3.06)	0.018	1.85 (1.11-3.07)	0.018	1.86 (1.12-3.10)	0.017
Asthma	1.58 (0.95-2.63)	0.078	1.59 (0.95-2.64)	0.076	1.58 (0.95-2.63)	0.077	1.58 (0.95-2.63)	0.078
No symptoms	1.00 (referene)		1.00 (referene)		1.00 (referene)		1.00 (referene)	
SPM (range: 26.7-42.7 µg/m^3^)	1.49 (1.07-2.06)	0.017	-		-		1.94 (1.08-3.50)	0.027
NO_2_ (range: 11.7-29.0 ppb)	-		1.41 (0.87-2.28)	0.164	-		0.62 (0.26-1.48)	0.287
SO_2_ (range: 4.3-6.3 ppb)	-		-		1.45 (1.04-2.03)	0.031	-	

Two-pollutant models including SO_2_ concentration were not analyzed, because the SO_2_ concentration very strongly correlated with SPM and NO_2_ (correlation coefficients = 0.99 and 0.92, respectively).

*: ORs were adjusted for all variables using each logistic regression model

SPM: Suspended particulate matter

NO_2_: Nitrogen dioxide

SO_2_: Sulfur dioxide

ppb: parts per billion

In models without respiratory symptoms and /or serum IgE concentration, the results were essentially the same (data not shown).

## DISCUSSION

CRP is one of the major acute-phase proteins in humans. It is produced mainly by the liver, and its production is regulated by cytokines, including interleukin-6.^9^ The production of CRP is enhanced when the complement system is activated by inflammatory reactions that accompany bacterial infections or tissue injury *in vivo*.^24^ Therefore, serum CRP concentration has frequently been used as a marker for inflammation.^9^

The author and coworkers previously reported that the serum concentrations of the acute-phase proteins C3c and C4 in children reflect their exposure level to air pollutants.^8^ In addition, the serum C3c concentration in boys is significantly increased by exposure to environmental tobacco smoke.^25^ In these previous studies, children with symptoms of a cold or fever were excluded to eliminate the effects of acute inflammation. Therefore, the serum CRP concentration in most children was below the detection level of 3.0 mg/L. A newly available assay for measuring CRP concentration has a detection level of 0.2 mg/dL, and has been found to be useful for early detection of infectious disease in newborns and immature infants.^9^ This serum CRP assay is considered to be particularly useful for evaluation of mild chronic inflammation in apparently healthy individuals.^11^^,^^24^

In the present study, the author conducted a cross-sectional study in 3 communities with different concentrations of air pollutants, and examined the serum CRP concentration in children. Serum CRP concentration differed significantly according to age, feeding method in infancy, maternal smoking habit, and wheezing. There were differences in feeding method in infancy and maternal smoking habit among the study communities. Multiple logistic regression analyses adjusted for these potential confounding factors showed that serum CRP concentrations of the 90th percentile or above were significantly associated with SPM concentration in each community. These findings suggest that air pollution affects serum CRP concentration in children, and are consistent with the results of previous reports showing an association between air pollution and concentrations of serum acute-phase proteins.^8^ Children with signs of acute inflammation were excluded from the present study, eliminating the possibility that reported differences were due to an epidemic of inflammatory diseases. In multivariate models without respiratory symptoms and/or serum IgE concentration, the associations between air pollutants and serum CRP concentration were essentially the same.

Serum CRP concentration is associated with factors such as age, obesity, and smoking habit.^24^^,^^26^ In chronic inflammation, a slight elevation in serum CRP concentration can persist.^9^ Therefore, serum CRP has been considered a useful marker for prediction of cardiac infarction or for evaluation of inflammation of blood vessels due to atherosclerosis in adults.^12^ A study in elderly people showed that serum CRP concentration increases with age, because older people are subject to the effects of low grade inflammation due to chronic disease.^26^ Adults with serum CRP concentrations of 3 mg/L or above are considered at high risk for cardiovascular diseases.^27^ The serum CRP concentration is reportedly lower in children than adults.^28^ In the present study, the concentrations were then dichotomized at the 90th percentile (1.4 mg/L). Serum CRP concentration was highest in the youngest children, and decreased with age among school children under 12 years old. Because younger children easily contract respiratory infections,^29^ low grade inflammation due to subclinical respiratory conditions may influence serum CRP concentration in young children.

The serum CRP concentration in children with wheeze was significantly higher than in children without wheeze. A population-based study in Finland reported that the prevalence of asthma increased gradually with increasing serum CRP concentration.^10^ Inflammation in the respiratory tract is thought to play an important role in the pathological conditions of wheezing and asthma.^30^ Wheezing is often caused by a viral infection.^31^ In the present study, the serum CRP concentration was slightly higher in children with asthma than those without, but the difference was not significant, possibly because most asthmatic children were asymptomatic when examined. An allergic predisposition, accompanied by increases in serum IgE concentration, is thought to be important for the development of the pathological states of childhood asthma. However, the author believes that no direct relationship exists between allergies and serum CRP concentration, because no association was found between serum IgE and CRP concentration.

The serum CRP concentration in children with mothers who smoked was significantly higher than those in children with non-smoking mothers. The serum CRP concentration in smokers is higher than in non-smokers.^32^ The author previously reported that concentrations of acute-phase proteins in children were elevated upon exposure to environmental tobacco smoke, and suggested the possibility that passive smoking may lead to slight inflammation in children.^25^ In the present study, the effects of smoking by family members other than mothers, i.e., mainly fathers, were not detected. This may reflect the lack of opportunity for fathers to smoke when with their children.

The complement activities of children who were bottle-fed in infancy have been reported to be high.^33^ Children who were both bottle- and breast-fed have been reported to have higher CRP concentrations than those who were only breast-fed.^34^ In the present study, children who were only bottle-fed had higher serum CRP concentrations than those who were only breast-fed or those who received mixed-feeding. The reason for this is not clear, but might be related to the protective effects of breast-feeding against infection during infancy.^35^

No significant difference in serum CRP concentration according to sex was observed. A study based on 3605 subjects found no differences in serum CRP concentration by sex between 5-39 years of age.^26^ Cook et al^34^ determined a relationship between serum CRP concentration in children and adiposity and suggested an association between CRP and cardiovascular disease. The involvement of serum CRP concentration with various factors such as sex, adiposity, body constitution, and symptoms other than respiratory symptoms should be further evaluated.

Air pollution due to automobile exhaust is a serious problem in the urban areas of Japan. The effect of fine particles in diesel exhaust on human health has become a matter of concern.^3^^,^^36^ Recently, air quality standards for atmospheric concentration of particulate matter less than 2.5 µm in diameter (PM_2.5_) have been established in the United States and European countries.^37^ Because atmospheric concentrations of PM_2.5_ have been rarely measured in Japan,^38^ the effects of SPM, NO_2_, and SO_2_ were examined in the present study. When various confounding factors were adjusted for, increased serum CRP concentrations of the 90th percentile or above were significantly associated with SPM and SO_2_ concentration. The adjusted OR of high serum CRP concentrations for the range of observed NO_2_ concentrations was similar to that for SPM and SO_2_, but this was not significant. In a two-pollutant model including both SPM and NO_2_ concentrations, high serum CRP concentration was associated only with SPM. These results suggest that SPM in the atmosphere may induce an inflammatory response in children more readily than NO_2_. However, the concentrations of SPM and NO_2_ are correlated, and the concentration of SO_2_ showed stronger correlation with these air pollutants. Therefore, it is difficult to elaborate on which pollutant has a stronger effect on serum CRP concentration. In addition, the effects of specific characteristics of the communities where the children lived cannot be completely eliminated.

A slight elevation in serum CRP concentration is thought to reflect chronic inflammation *in vivo*.^39^ Acute effects of particulate air pollution on serum CRP concentration have been reported in middle-aged or elderly subjects,^16^^,^^18^^,^^40^ and in individuals with diabetes, obesity, hypertension, and coronary heart disease.^15^^,^^17^ Recently, an acute effect of particulate air pollution on serum CRP was also observed in healthy young people.^19^ A weak positive association between CRP and 60-day mean exposure to PM_2.5_ has been also reported.^14^ Because the concentrations of air pollutants did not change greatly during the study period, the chronic effect of air pollution on serum CRP concentration was examined using 3-year average concentrations of air pollutants. Induction of proinflammatory cytokines and CRP has also been reported in macrophage cell lines exposed to ambient air particulates.^41^^,^^42^ Particulate air pollution may cause slight chronic inflammation in the respiratory tract in children,^4^ and may induce an inflammatory response in humans more readily than NO_2_. The pathophysiological significance of changes in serum CRP concentration is unknown, but is a topic worthy of additional study.

In conclusion, high serum CRP concentrations of the 90th percentile or above are associated with atmospheric concentrations of SPM. These findings suggest that air pollutants such as SPM may cause a slight inflammation *in vivo*. Slight elevations in serum CRP concentration, such as those observed in the present study, are thought to be a risk factor for cardiovascular disease and have been associated with particulate air pollution.
